# Association between patterns of nutrient intake and circulating vitamin D with sleep status among Iranian adults

**DOI:** 10.1038/s41598-023-42661-6

**Published:** 2023-09-15

**Authors:** Parisa Rouhani, Keyhan Lotfi, Javad Anjom-Shoae, Zahra Hajhashemi, Elahe Mokhtari, Zahra Heidari, Parvane Saneei

**Affiliations:** 1 Students’ Research Committee, Isfahan University of Medical Sciences, Isfahan, Iran; 2 Department of Community Nutrition, School of Nutrition and Food Science, Nutrition and Food Security Research Center, Isfahan University of Medical Sciences, PO Box 81745-151, Isfahan, Iran; 3 Department of Community Nutrition, School of Nutritional Sciences and Dietetics, Tehran University of Medical Sciences, Tehran, Iran; 4 Adelaide Medical School, Faculty of Health and Medical Sciences, University of Adelaide, Adelaide, Australia; 5 Department of Biostatistics and Epidemiology, School of Health, Isfahan University of Medical Sciences, Isfahan, Iran

**Keywords:** Nutrition, Public health

## Abstract

Nutrient pattern analysis is an easy way to compare nutrient intakes across different nations due to the universality of nutrients nature. The purpose of this study was to examine the relationship between dietary nutrient patterns (NPs) and circulating 25(OH)D concentrations with sleep duration and sleep quality among Iranian adults. We used a multistage cluster random sampling method to enroll 535 adults in this cross-sectional investigation. A validated food frequency questionnaire was applied to evaluate typical dietary intakes. Fasting blood samples were obtained to determine levels of circulating 25(OH)D. Sleep characteristics were assessed using the Pittsburgh Sleep Quality Index (PSQI). Participants had a mean age of 42.57 years and 51.2% of them had insufficient or deficient levels of serum vitamin D. Three NPs were identified: "high animal protein", "high vegetable" and "high carbohydrate". After adjustments for potential confounders, no significant associations were observed between "high animal protein" pattern and short sleeping or sleep quality. Greater adherence to "high vegetable" NP was associated with lower odds of short sleeping (OR 0.24; 95% CI 0.10, 0.54) and poor sleep quality (OR 0.45; 95% CI 0.20, 1.05). Stratified analysis revealed that these associations were stronger in normal-weight participants. Greater adherence to "high carbohydrate" NP, on the other hand, was connected to higher odds of short sleeping (OR 2.83; 95% CI 1.20, 6.72). Low adherence to "high vegetable" pattern and vitamin D insufficiency/deficiency were jointly associated with increased odds of short sleeping (OR 3.42, 95% CI 1.42, 6.64). High adherence to pattern comprising mainly of vegetable nutrients was associated with a reduced likelihood of being short sleepers and having poor sleep quality in Iranian adults, especially among those with a normal weight. Lower adherence to vegetable NP and insufficient/deficient vitamin D levels were synergistically associated with greater likelihood of being short sleepers. Greater adherence to carbohydrate NP was associated with an increased likelihood of short sleeping.

## Introduction

Sleep is of the great essence in the human daily routine which accounts for one-third part of the lifetime^1^. The National Sleep Foundation (NSF) advises 7–9 h of daily sleep for adults younger than 65 years and 7–8 h for older adults, although sleep needs may vary by both age and gender^2^. In recent decades, sleep disorders have become a worldwide pandemic^3,4^, but many individuals are unaware of the importance of their sleep habits. According to studies, the global prevalence of sleep disorders ranged from 1.6 to 56%^5,6^, with an increasing rate in most populations^7,8^. Numerous common negative outcomes have been associated with sleep difficulties. Sleep durations of more or less than 7–9 h in a day are associated with cardiovascular diseases (CVD)^9^, diabetes^10^, obesity^10,11^, work failures^12^, learning or memory problems^13^ and high mortality rate^14–16^. Also, an appropriate sleep quality is a crucial item in physiologic recovery of the body, and preventing CVD^17^, depression, anxiety^18^, and obesity^19^.

Several factors, such as physical activity, individual dietary elements, nutrients, and even covid-19 pandemic, have been discovered to affect sleep duration and quality^20–23^. Some studies revealed that diets with low fruits and vegetables^24^, and high sweets or unhealthy fats have been connected to poor sleep quality^25,26^. Among nutrients, vitamin D is a unique, fat-soluble vitamin which can be obtained either by diet or ultraviolet-B (UV-B) radiation synthesis^27^. Several factors (such as lifestyle, sunlight exposure, skin color, and sex hormones) are involved in vitamin D deficiency (VDD), which is prevalent worldwide^27,28^. Although the associations of VDD with CVD^29^, infectious diseases^30^, and sleep disorders^31^ have recently received considerable attention, all functions of vitamin D in the body have not been well-recognized. Low vitamin D levels have been reported to be associated with shorter sleep duration^32^. The expression of vitamin D receptors (VDRs) in areas of the brainstem might be involved in sleep regulation^33,34^. VDRs are expressed in the cortical and subcortical areas involved in sleep control^35^.

People consume several nutrients together, which might interact with each other^36^. Nutrients can also affect the absorption and function of one another in a synergistic manner. Considering nutrient intake patterns has a number of advantages over dietary patterns. Nutrients are consumed globally, whereas the consumption of foods varies by geographic region and culture^37^. Consequently, nutrient pattern (NP) analysis provides a more accurate depiction of diet-disease relationships than dietary pattern analysis, especially when comparing dietary intakes across nations. In NP analysis, unlike food patterns, the underlying mechanisms on the diet–disease associations can be explained by nutrient content of patterns. Also, NP analysis can be an interface between food patterns and food metabolome integrating the measurements of both diet and metabolism^37,38^.

The vast majority of previous research on the association between dietary intakes and sleep duration and quality in adults has focused on particular foods, food groups, dietary patterns^39–42^; some others have focused on dietary habits such as timing of food intake and chronotypes related to dietary intakes or behaviors^43^. In addition, a previous study has reported that different dietary NPs were associated with sleep time and sleep latency in Iranian adults with overweight and obesity^44^. However, the association between circulating vitamin D levels and sleep disorders was not investigated among Iranian adults. The results of previous studies that investigated this association in other populations were also controversial^45–47^. Consequently, the purpose of this cross-sectional research was to examine the relationship between patterns of nutrient intake and circulating 25(OH)D concentrations with sleep duration and quality among Iranian adults.

## Materials and methods

### Study design and participants

The current cross-sectional study was conducted on a relatively representative sample of adults from Isfahan city of Iran, in 2021. Considering a prevalence of 35% for lack of sufficient sleep duration and sleep quality among Iranian adults^48^, a confidence of 95%, and precision (d) of 4.1%, 519 subjects were approximately needed for this investigation. Data were not collected during COVID-19 waves or quarantine time. However, given the possibility of a low response rate due to the prevalence of the COVID-19 pandemic during data collection, 600 individuals were invited to participate in the study. Using a multistage cluster random sampling method, these middle-aged individuals (of both sexes) were randomly selected from 20 schools in Isfahan. To obtain a somehow representative sample of the general adult population with varying socioeconomic status, we included all adults employed in the chosen schools, including employees, teachers, school administrators, assistants, and crews. However, subjects with the following criteria were precluded from the study: (1) being pregnant or breastfeeding, (2) having a history of type 1 diabetes, CVD, stroke, or cancer, and (3) adhering to a weight-loss or weight-gain diet. Among invited subjects, 543 of them agreed to participate in our investigation (response rate: 90.5%). We did not also include individuals with the following criteria: (1) reported a total energy intake outside the range of 800–4200 kcal/day (as under-reporters and over-reporters of energy intake) (n = 3); and (2) had left more than 70 items blank on the food frequency questionnaire (n = 4); (3) did not accept blood draw. At the end, 535 adults were included in this analysis (Supplementary Fig. S1).

### Assessment of dietary intakes

A validated Willett-format semi-quantitative 168-item food frequency questionnaire (FFQ) was applied to assess usual dietary intakes of participants^49^. A previous validation study of this FFQ on 132 middle-aged adults revealed significant correlations between dietary intakes assessed by this FFQ and those obtained from multiple 24-h dietary recalls^49^. The correlation coefficients were 0.55 for total energy, 0.65 for proteins, 0.59 for fat, 0.67 for fiber, and 0.65 for magnesium. The reproducibility of the FFQ was determined by comparing nutrient intakes derived from the FFQ on two separate occasions separated one year apart. Overall, these findings indicated that this tool could provide reasonably valid and reliable measures for dietary intakes of Iranian adults^49^. An expert dietitian instructed the study participants to complete the FFQ by reporting the frequency and quantity of consumed food items over the previous year. Then, using household measurements, all reported values were converted to gram per day^50^. Total energy and nutrient intakes of each individual were then calculated by summing up energy and nutrients of all food items. In addition, daily nutrients intake for each participant was calculated the according to the nutrient contents of all foods. Then, in order to determine daily energy and nutrient intake, all food items were entered into the Nutritionist IV software whose nutrient database was based on USDA food composition table, but was modified for some traditional Iranian foods.

### Biochemical assessment

After 12 h of fasting, blood samples were collected via venipuncture, then aliquoted and centrifuged immediately. After transporting all serum samples to the central laboratory on dry ice, they were kept at −80 °C until analysis. Using an ELISA commercial kit (Monobined Inc. Lake Forest, CA 92630, USA), serum 25(OH)D levels were measured.

### Assessment of sleep habits

The Pittsburgh Sleep Quality Index (PSQI) questionnaire was used to evaluate measures of habitual sleep quality and quantity^51^; the validity and reliability of this tool have been previously published^52^. The PSQI is conducted to evaluate patterns of sleep over the previous month and can be answered alone or with a sleeping partner. This questionnaire presents a complex score (ranged 0–21), pointing to total sleep quality. Every single item is weighted from zero to three (0 = good vs. 3 = poor). At final, the scores of the items are summed to get a total score of 0 to 21. Higher scores of complex PSQI suggests lower sleep quality. This measurement tool also includes seven domains evaluating: (1) person's general description of his/her sleep; (2) delay in falling asleep; (3) duration of beneficial sleep; (4) adequacy of sleep (calculated based on ratio of duration of valuable sleep to total time spent for sleep), (5) sleep disorders (in form of waking up at night), (6) use of sleeping medicine, and (7) morning performance in form of problems experienced by the person during the day due to poor sleep. In the current analysis, the overall scores of PSQI were applied to group subjects into total sleep quality as a dichotomous variable: poor (score ≥ 6) vs. good (score < 6)^51^. Sleep quantity or duration was the second exposure of interest for this analysis, which was defined as the time of sleep duration per night. Individuals with less than 6 h sleep at night were considered as short sleepers.

### Other variable assessment

Height and weight of subjects were measured while they were standing in minimal clothes and barefoot. The height was measured to the nearest 0.1 cm using a measuring instrument. A body composition analyzer (Tanita MC-780MA, Tokyo, Japan) was used to measure weight. Body mass index (BMI) was determined by dividing weight (kg) by height squared (m^2^). After 5 min of rest, blood pressure was measured twice in a seated position using a digital sphygmomanometer (OMRON, M3, HEM-7154-E, Japan) with an accuracy of 0.5 mmHg; the mean of these measurements was recorded for each participant. Data of additional confounders such as age, sex, marital status, education, smoking habits, socio-economic status, medical history of diseases, medication use, and other confounders were gathered through pre-tested questionnaires.

### Statistical analysis

The Kolmogorov–Smirnov test was applied to examine normality of quantitative variables. Mean ± SD/SE and number (percentage) were respectively provided for continuous and categorical variables. Major NPs were identified by factor analysis using thirty-five nutrients and bioactive compounds. Varimax rotation was applied to improve interpretability and* minimize the correlation between the identified factors. The Kaiser–Meyer–Olkin (KMO) test was used to determine if distribution of different nutrients allowed the use of principal components. On the basis of Eigen values (≥ 2), Scree plot, and interpretability of the factors, the number of main factors was determined. Each nutrient's factor loadings were determined by factor analysis. Factor scores for each NP were additionally derived by adding the total grams of all nutrients, weighted by their factor loadings by factor analysis. Then, we labeled NPs, based on nutrient groups that loaded high in each pattern. Participants were divided into quartiles of NP scores. One-way ANOVA and chi-square tests were used to investigate the differences in quantitative and qualitative variables across quartiles of major NPs. Binary logistic regression was used to have OR and 95% CI for short sleep duration (< 6 vs. ≥ 6 h) and poor sleep quality (score ≥ 6 vs. < 6) across quartiles of major NPs. Based on previous literature^53–40^, age, sex, and energy intake were adjusted in the first model. Additional adjustments were done for marital status, physical activity, and family size, history of diabetes, hypertension, smoking status, education status, obstructive sleep apnea (OSA), anxiety, distress, home ownership, anti-depressing medication, tea and coffee consumption (as the main sources of caffeine) in the second model. In the last model, further adjustment for BMI was done. Participants in the first quartile of major NPs were considered as the reference category in all models. Quartiles of each NP were treated as an ordinal variable in order to test the trend. SPSS version 20 was applied to conduct all statistical analyses. P values were considered significant at < 0.05.

### Ethical approval and consent to participate

All procedures were conducted in accordance with STROBE regulations and guidelines. All procedures were carried out in accordance with applicable rules and guidelines. Each participant signed a written informed consent form. The study protocol was ethically approved by Isfahan University of Medical Sciences (no. 1400461).

## Results

The average age and BMI of all participants of this study (n = 535) were 42.57 (years) and 26.91 (kg/m^2^), respectively. Among them, 46% were female. Table 1 illustrates three major NPs identified among the study subjects. Factor 1 (NP1), which contained a high intake of animal protein, cobalamin, zinc, saturated fatty acid (SFA), phosphorus, riboflavin, cholesterol, mono-unsaturated fatty acids (MUFAs), calcium, pantothenic acid, was labeled as "high animal protein". Factor 2 (NP2), which contained a high intake of fiber, potassium, vitamin A, C, K, trans fatty acids (TFAs), magnesium, folate, pyridoxine, and copper, was labeled as "high vegetable". Factor 3 (NP3), which was characterized by high intake of thiamin, plant protein, selenium, iron, niacin and carbohydrate, was named as "high carbohydrate". These 3 factors explained 65.16% of total variance of nutrient intake. The KMO coefficient was 0.88, indicating adequate sampling.

**Table 1:** Factor loadings and explained variances for major nutrient patterns (NPs).

	Factor loadings		
	NP1 High animal protein	NP2 High vegetable	NP3High carbohydrate
Animal protein (gr)	0.92	–	–
Cobalamin (mcg)	0.91	–	–
Zinc (mg)	0.86	0.30	–
SFA (gr)	0.84	–	–
Phosphorus (mg)	0.83	0.39	–
Riboflavin (mg)	0.79	0.423	–
Cholesterol (mg)	0.78	–	–
MUFA (gr)	0.77	–	0.31
Calcium (mg)	0.73	0.37	–
Pantothenic acid (mg)	0.71	0.54	0.25
PUFA (gr)	0.50	–	0.32
Sodium (mg)	0.35	0.20	–
Vitamin D (mcg)	0.34	–	0.27
Total fiber (gr)	–	0.89	0.58
Vitamin C (mg)	–	0.87	–
Potassium (mg)	0.25	0.85	–
TFA (gr)	–	0.77	–
Folate (mcg)	0.39	0.74	0.30
Vitamin A (RE)	0.24	0.72	–
Magnesium (mg)	0.55	0.72	0.29
Pyridoxine (mg)	0.45	0.67	0.36
Vitamin K (mcg)	0.23	0.66	–
Copper (mg)	0.42	0.66	0.46
Vitamin E (mg)	0.31	0.51	0.33
Manganese (mg)	–	0.49	0.37
Biotin (mcg)	0.36	0.46	0.42
Fluoride (mcg)	–	0.20	–
Thiamin (mg)	0.21	0.36	0.84
Plant protein (gr)	–	0.41	0.77
Selenium (mg)	–	–	0.75
Iron (mg)	0.20	0.32	0.74
Niacin (mg)	0.45	0.26	0.73
Chromium (mg)	–	–	0.69
Carbohydrate (gr)	–	0.55	0.67
Sugar (gr)	–	–	0.24
Variance explained (%)	25.57	23.82	15.77
Cumulative explained variance (%)	25.57	49.39	65.16

^1^Factor loadings <|0.20| are not shown for simplicity. The Kaiser–Meyer–Olkin value was 0.88. Factors with Eigen values ≥ 2 were used to extract major NPs.

General features of individuals across quartiles of major NPs are depicted in Table 2. In comparison to the lowest quartile of NP1, participants in the highest quartile had higher weight, coffee consumption and lower age and tea drinking. In terms of NP2, participants in the top quartile had higher age, coffee and tea intake, and lower height, distress score and were less likely to be male. In comparison to those in the lowest quartile of NP3, participants in the highest quartile had more tea drinking, height, weight, and were more likely to be male.

**Table 2:** General characteristics factors of study participants across quartiles of major nutrient patterns.

	Quartiles of NP1 High animal protein		P-value^1^	Quartiles of NP2 High vegetable		P-value^1^	Quartiles of NP^3^ High carbohydrate		P-value^1^
	Q1 (n = 132)	Q4 (n = 133)		Q1 (n = 133)	Q4 (n = 135)		Q1 (n = 132)	Q4 (n = 133)	
Age (years)	45.39 ± 11.24	40.77 ± 9.81	< 0.001	40.35 ± 10.18	45.81 ± 11.14	< 0.001	43.63 ± 11.84	40.82 ± 11.99	0.187
Height (cm)	166.43 ± 43	168.96 ± 8.86	0.10	168.94 ± 8.96	165.99 ± 7.58	0.01	165.90 ± 7.53	170.01 ± 8.46	< 0.001
Weight (kg)	73.81 ± 14.94	77.89 ± 15.40	0.04	76.90 ± 15.77	74.63 ± 14.24	0.38	72.68 ± 12.29	79.06 ± 16.81	< 0.001
Marital status (Married), n (%)	106 (80.9)	114 (85.7)	0.57	111 (84.7%)	111 (82.8)	0.57	113 (86.9)	107 (81.1)	0.24
Body mass index (kg/m^2^)	26.63 ± 4.79	27.16 ± 4.26	0.37	26.82 ± 4.40	27.07 ± 4.79	0.95	26.42 ± 4.09	27.31 ± 5.22	0.23
Sex (Male), n (%)	74 (56.1)	71 (53.4)	0.95	81 (60.9)	62 (45.9)	0.04	54 (40.9)	97 (72.9)	< 0.001
Physical activity (Inactive), n (%)	80 (60.6)	68 (51.1)	0.25	78 (58.6)	76 (56.3)	0.89	79 (60.3)	71 (53.4)	0.62
OSA (Yes), n (%)	4 (3.2)	11 (8.8)	0.17	6 (4.9)	9 (6.8)	0.31	5 (3.9)	8 (6.3)	0.07
Smoking (Yes), n (%)	5 (4.3)	5 (4.1)	0.59	4 (3.3)	3 (2.5)	0.39	3 (2.5)	2 (1.7)	0.05
Coffee intake (mg/d)	9.34 ± 36.32	34.59 ± 80.50	0.008	15.56 ± 42.51	20.40 ± 55.13	0.002	22.36 ± 59.08	16.36 ± 50.58	0.70
Tea intake (mg/d)	830.59 ± 1077.85	709.84 ± 773.71	0.02	543.48 ± 437.46	940.66 ± 1222.59	< 0.001	532.72 ± 466.18	859.26 ± 1066	0.005
Family members (> 4), n (%)	18 (13.8)	16 (14.5)	0.29	17 (12.9)	20 (14.9)	0.92	15 (11.54)	19 (14.4)	0.27
House ownership (Yes), n (%)	89 (72.4)	106 (82.2)	0.17	93 (73.2)	105 (80.2)	0.28	32 (25.0)	31 (24.2)	0.99
Hypertension (Yes), n (%)	38 (29.2)	36 (27.5)	0.99	36 (27.9)	46 (34.3)	0.30	33 (25.4)	40 (30.3)	0.55
Antidepressant medicine use, n (Yes) (%)	11 (8.6)	7 (5.4)	0.24	7 (5.4)	11 (8.4)	0.27	9 (6.9)	6 (4.6)	0.81
History of type 2 diabetes (Yes), n (%)	8 (6.1)	6 (4.5)	0.68	6 (4.5)	11 (8.1)	0.37	8 (6.2)	4 (3.0)	0.39
Distress score	2.85 ± 2.56	2.3 ± 2.5	0.25	2.84 ± 2.65	2.00 ± 2.15	< 0.001	2.43 ± 2.62	2.47 ± 2.31	0.53
Anxiety score	2.22 ± 2.52	2.35 ± 2.72	0.87	2.11 ± 2.65	1.87 ± 1.97	0.18	2.06 ± 2.73	2.17	0.79
Education (University graduated), n (%)	111 (84.7)	118 (89.4)	0.18	120 (90.2)	115 (87.1)	0.66	118 (90.1)	114 (85.7)	0.31

Values are Mean ± SD; unless indicated.

*kg* kilogram, *m* meter, *cm* centimeter, *BMI* Body Mass Index, *n* number, *mmHg* millimeter, *OSA* obstructive sleep apnea.

^1^Obtained from ANOVA and χ2 test for quantitative and categorical variables, respectively.

Table 3 provides daily dietary intakes of individuals across quartiles of major NPs. Adults in the top quartile of NP1 had higher intake of energy, animal protein, fat, SFA, calcium, white meat, dairy, red and processed meat, and lower consumption of carbohydrate, vitamin C, iron, fiber, fruits, legume, whole and refined grains in comparison to the those in the lowest category. In case of NP2, participants in the highest quartile, compared to individuals in the lowest one, had more consumption of energy, animal and plant protein, carbohydrate, vitamin C, E, pyridoxine, folate, calcium, fiber, vegetables, fruits, and legume and lower consumption of fat, SFA, iron, red and processed meat, whole and refined grain. Compared with individuals in the lowest quartile of NP3, those in the highest quartile had higher consumption of energy, plant protein, carbohydrate, iron, and whole grains, and lower intake of fat, SFA, vitamin C, pyridoxine, calcium, fiber, sodium, vegetable, fruits, dairy, refined grain, and white meat.

**Table 3:** Daily dietary intakes of study participants across quartiles of major nutrient patterns (NPs).

	Quartiles of NP1 High animal protein			Quartiles of NP2 High vegetable			Quartiles of NP3 High carbohydrate		
	Q1 (n = 132)	Q4 (n = 133)	P-value^1^	Q1 (n = 133)	Q4 (n = 135)	P-value^1^	Q1 (n = 132)	Q4 (n = 133)	P-value^1^
Energy, kcal/d	1842.39 ± 50.87	2818.39 ± 50.44	< 0.001	1914.16 ± 52.75	2722.29 ± 52.77	< 0.001	1733.71 ± 48.02	2867.48 ± 48.49	< 0.001
Carbohydrate, % of E	68.99 ± 0.51	53.00 ± 0.50	< 0.001	57.87 ± 0.69	64.56 ± 0.69	< 0.001	58.60 ± 0.71	63.33 ± 0.71	< 0.001
Fat, % of E	20.75 ± 0.46	32.37 ± 0.46	< 0.001	28.01 ± 0.58	24.87 ± 0.58	0.001	28.40 ± 0.59	25.01 ± 0.59	0.001
Animal protein, gr/d	22.98 ± 1.10	76.28 ± 1.09	< 0.001	43.68 ± 2.00	51.45 ± 2.00	0.003	43.72 ± 2.03	47.60 ± 2.05	0.21
Plant protein, gr/d	33.07 ± 1.07	36.91 ± 1.07	0.04	29.05 ± 0.99	41.39 ± 0.99	< 0.001	22.61 ± 0.71	48.70 ± 0.72	< 0.001
Saturated fatty acids, gr/d	14.81 ± 0.55	31.53 ± 0.57	< 0.001	25.23 ± 0.69	19.21 ± 0.71	< 0.001	27.75 ± 0.69	15.78 ± 0.72	< 0.001
Vitamin C, mg/d	220.14 ± 9.29	173.17 ± 9.56	0.009	107.13 ± 6.33	326.84 ± 6.48	< 0.001	257.46 ± 9.31	143.89 ± 9.58	< 0.001
Pyridoxine, mg/d	1.73 ± 0.05	1.80 ± 0.05	0.25	1.45 ± 0.04	2.22 ± 0.04	< 0.001	1.98 ± 0.05	1.67 ± 0.05	0.001
Vitamin E, mg/d	7.28 ± 0.29	6.92 ± 0.30	0.25	5.64 ± 0.27	8.16 ± 0.28	< 0.001	7.30 ± 0.30	6.50 ± 0.31	0.36
Folate, mcg/d	340.85 ± 10.48	347.68 ± 10.78	0.25	254.95 ± 8.48	449.38 ± 8.68	< 0.001	367.19 ± 10.91	323.70 ± 11.23	0.06
Iron, mg/d	20.45 ± 0.35	15.46 ± 0.36	< 0.001	18.81 ± 0.37	17.49 ± 0.37	0.02	14.72 ± 0.34	21.81 ± 0.35	< 0.001
Calcium, mg/d	696.70 ± 30.44	1256.07 ± 31.33	< 0.001	872.01 ± 33.61	1010.83 ± 34.40	0.03	1228.21 ± 32.11	603.44 ± 33.05	< 0.001
Total fiber, gr/d	23.84 ± 0.58	17.59 ± 0.59	< 0.001	14.82 ± 0.40	29.46 ± 0.41	< 0.001	22.74 ± 0.62	19.55 ± 0.64	0.01
Sodium, mg/d	3380.65 ± 235.47	4038.93 ± 242.35	0.09	3649.06 ± 230.95	3694.57 ± 236.39	0.79	4283.70 ± 245.30	2979.35 ± 252.50	0.006
Food groups									
Vegetables, g/d	357.51 ± 21.48	342.72 ± 22.10	0.64	207.24 ± 17.96	561.47 ± 18.38	< 0.001	405.08 ± 22.24	270.97 ± 22.89	0.002
Fruits, g/d	662.60 ± 28.64	419.50 ± 29.48	< 0.001	295.60 ± 22.68	899.32 ± 23.21	< 0.001	737.33 ± 29.15	361.17 ± 30.01	< 0.001
Dairy, g/d	151.89 ± 21.31	567.05 ± 21.94	< 0.001	300.24 ± 24.04	339.94 ± 24.61	0.67	502.25 ± 23.40	102.77 ± 24.09	< 0.001
Whole grains, g/d	150.03 ± 7.16	81.31 ± 7.37	< 0.001	146.33 ± 7.03	83.88 ± 7.20	< 0.001	65.84 ± 7.26	163.71 ± 7.48	< 0.001
Refined grain, g/d	333.20 ± 14.48	201.97 ± 14.91	< 0.001	313.75 ± 14.22	206.79 ± 14.55	< 0.001	138.76 ± 13.02	450.51 ± 13.40	< 0.001
Legume, g/d	38.28 ± 3.38	32.86 ± 3.47	0.01	27.56 ± 3.27	41.20 ± 3.35	< 0.001	34.55 ± 3.56	46.31 ± 3.66	0.18
Nuts, g/d	9.84 ± 1.18	13.55 ± 1.21	0.20	9.23 ± 1.14	12.90 ± 1.17	0.72	11.65 ± 1.23	10.66 ± 1.27	0.49
Red and processed meat, g/d	38.80 ± 3.75	104.34 ± 3.86	< 0.001	85.97 ± 3.98	52.43 ± 4.08	< 0.001	74.78 ± 4.38	59.13 ± 4.51	0.13
White meat, g/d	22.85 ± 2.92	56.87 ± 3.01	< 0.001	42.32 ± 3.01	35.44 ± 3.08	0.46	44.21 ± 3.21	30.01 ± 3.30	0.03

Values are Mean ± SE. Energy intake and carbohydrates and fats were adjusted for age and sex; all other values were adjusted for age, sex and energy intake.

*E* energy intake, *g* gram, *mg* milligram, *mcg* microgram.

^1^Obtained from ANCOVA.

**Figure 1:**
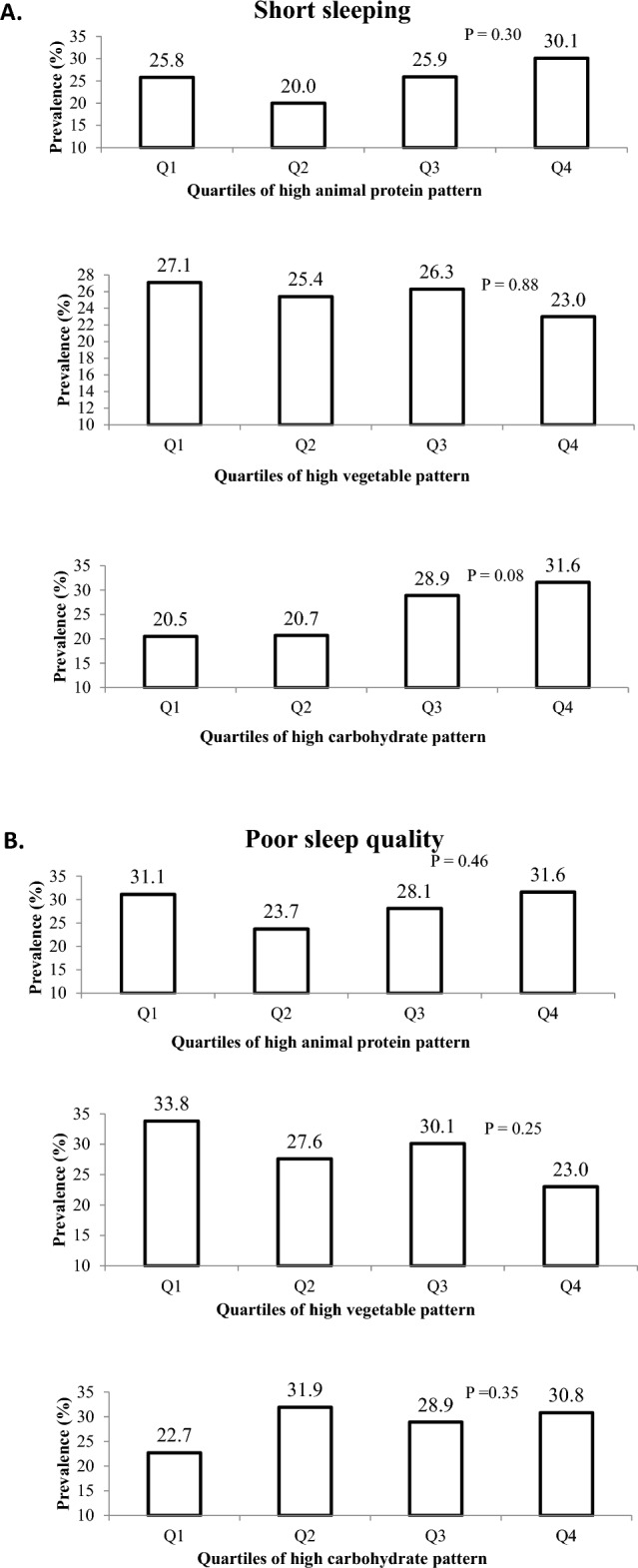
(**A**) Prevalence of short sleeping across quartiles of major nutrient patterns (NPs). (**B**) Prevalence of having poor sleep quality across quartiles of major nutrient patterns (NPs).

Prevalence of participants with short sleeping and poor sleep quality across quartiles of major NPs is illustrated in Fig. 1. Prevalence of short sleepers (< 6 h/night) in the top quartile of NP3 was slightly higher in comparison to the bottom quartile (31.6 vs. 20.5%); this difference was marginally significant (P = 0.08). Moreover, individuals in the highest category of NP2 had a non-significant lower prevalence of poor sleep quality (23.0 vs. 33.8%, P = 0.25).

Crude and multivariable-adjusted ORs and 95% confidence intervals (CIs) for short sleeping and poor sleep quality across quartiles of major NPs are illustrated in Table 4. Participants with the highest adherence to NP2 (high vegetable) compared to the lowest one had 51% lower odds of being short sleepers (< 6 h per night) in the first model (OR 0.49, 95% CI 0.26, 0.92). After adjustments of other potential confounders, this relation was strengthened (OR 0.24, 95% CI 0.10, 0.54). Also, a significant decreasing trend (P_trend_ < 0.001) was observed for the odds of being short sleepers across quartiles of NP2. A significant inverse relation was observed between adherence to the NP2 and poor sleep quality in crude model (OR 0.58, 95% CI 0.34, 0.99). Considering potential confounders, individuals with the highest adherence to NP2 had a marginally significant lower odds of having poor sleep quality (OR 0.45, 95% CI 0.20, 1.05). After taking potential confounders into account, greater adherence to "high carbohydrate" NP (NP3) was connected to higher odds of short sleeping (OR _Q4 vs. Q1_ = 2.83; 95% CI 1.20, 6.72). No other significant relation was found between NP1 and NP3 with sleep duration or sleep quality.

**Table 4:** Multivariate adjusted odds ratio (OR) and 95% confidence interval (CI) for short sleeping and poor quality of sleep across quartiles of major nutrient patterns (NPs).

	Quartiles of NP1			High protein	
	Q1 (n = 132)	Q2 (n = 135)	Q3 (n = 135)	Q4 (n = 133)	P-trend^1^
Short sleeping					
Cases (n)	34	27	35	40	
Crude	1 (Ref.)	0.72 (0.41, 1.28)	1.01 (0.58, 1.75)	1.24 (0.72, 2.12)	0.26
Model 1	1 (Ref.)	0.68 (0.38, 1.22)	0.95 (0.53, 1.69)	1.00 (0.53, 1.89)	0.76
Model 2	1 (Ref.)	0.58 (0.27, 1.24)	1.02 (0.48, 2.15)	1.31 (0.58, 2.96)	0.22
Model 3	1 (Ref.)	0.58 (0.27, 1.25)	1.01 (0.48, 2.15)	1.31 (0.58, 2.96)	0.23
Poor sleep quality					
Cases (n)	41	32	38	42	
Crude	1 (Ref.)	0.69 (0.40, 1.19)	0.87 (0.51, 1.47)	1.02 (0.61, 1.72)	0.73
Model 1	1 (Ref.)	0.66 (0.38, 1.14)	0.81 (0.64, 1.42)	0.92 (0.49, 1.70)	0.92
Model 2	1 (Ref.)	0.55 (0.26, 1.20)	0.68 (0.31, 1.48)	0.80 (0.33, 1.93)	0.82
Model 3	1 (Ref.)	0.54 (0.25, 1.18)	0.67 (0.31, 1.48)	0.80 (0.33, 1.93)	0.83

	Quartiles of NP2			High vegetable	
	Q1 (n = 133)	Q2 (n = 134)	Q3 (n = 133)	Q4 (n = 135)	P-trend^1^
Short sleeping					
Cases (n)	36	34	35	31	
Crude	1 (Ref.)	0.92 (0.53, 1.58)	0.96 (0.56, 1.66)	0.80 (0.46, 1.40)	0.50
Model 1	1 (Ref.)	0.86 (0.49, 1.49)	0.73 (0.41, 1.29)	0.49 (0.26, 0.92)	0.03
Model 2	1 (Ref.)	0.67 (0.35, 1.31)	0.39 (0.19, 0.81)	0.24 (0.10, 0.54)	< 0.001
Model 3	1 (Ref.)	0.67 (0.34, 1.30)	0.39 (0.19, 0.82)	0.24 (0.10, 0.54)	< 0.001
Poor sleep quality					
Cases (n)	45	37	40	31	
Crude	1 (Ref.)	0.75 (0.44, 1.26)	0.84 (0.50, 1.41)	0.58 (0.34, 0.99)	0.09
Model 1	1 (Ref.)	0.68 (0.40, 1.15)	0.72 (0.42, 1.25)	0.42 (0.23, 0.79)	0.01
Model 2	1 (Ref.)	0.55 (0.27, 1.12)	0.59 (0.28, 1.25)	0.46 (0.18, 1.06)	0.08
Model 3	1 (Ref.)	0.55 (0.27, 1.13)	0.59 (0.28, 1.24)	0.45 (0.20, 1.05)	0.08

	Quartiles of NP3			High carbohydrate	
	Q1 (n = 132)	Q2 (n = 135)	Q3 (n = 135)	Q4 (n = 133)	P-trend^2^
Short sleeping					
Cases (n)	27	28	39	42	
Crude	1 (Ref.)	1.02 (0.56, 1.84)	1.58 (0.90, 2.78)	1.80 (1.03, 3.14)	0.01
Model 1	1 (Ref.)	0.99 (0.54, 1.81)	1.47 (0.80, 2.73)	1.64 (0.81, 3.33)	0.01
Model 2	1 (Ref.)	1.29 (0.62, 2.68)	1.45 (0.66, 3.20)	2.87 (1.21, 6.78)	0.02
Model 3	1 (Ref.)	1.28 (0.62, 2.67)	1.42 (0.65, 3.14)	2.83 (1.20, 6.72)	0.02
Poor sleep quality					
Cases (n)	30	43	39	41	
Crude	1 (Ref.)	1.60 (0.92, 2.74)	1.38 (0.80, 2.40)	1.52 (0.88, 2.62)	0.23
Model 1	1 (Ref.)	1.70 (0.97, 2.98)	1.59 (0.87, 2.92)	2.00 (1.00, 4.02)	0.08
Model 2	1 (Ref.)	1.50 (0.72, 3.12)	1.03 (0.45, 2.35)	1.98 (0.79, 5.00)	0.29
Model 3	1 (Ref.)	1.51 (0.72, 3.15)	1.05 (0.46, 2.40)	2.01 (0.80, 5.09)	0.27

^1^All values are odds ratios and 95% confidence intervals. Model 1: Adjusted for age, sex, and energy intake. Model 2: Additionally adjusted for marital status, physical activity, homeownership, anti-depressing medication, education status, obstructive sleep apnea (OSA), distress, family size, history of hypertension, diabetes, smoking status, coffee and tea consumption, and anxiety; Fully adjusted model: More adjustments were done for body mass index (BMI).

^2^Obtained by the use of quartiles of major nutrient patterns as an ordinal variable in the model.

Multivariable-adjusted ORs and 95% CI for each domain of sleep quality across quartiles of major NPs are represented in Table 5. Participants with the highest adherence to NP3 (high carbohydrate) were 4.55 times more likely to have daily functional disorders in fully-adjusted model (OR 4.55, 95% CI 1.09, 18.91). No other significant relation was observed between adherence to NPs and different sleep quality domains, after taking all potential confounders into account.

**Table 5:** Multivariate adjusted odds ratio (OR) and 95% confidence interval (CI) for individual domains of sleep quality across quartiles of major nutrient patterns (NPs).

	Quartiles of NP1 High protein			Quartiles of NP2 High vegetable			Quartiles of NP3 High carbohydrate		
	Q1	Q4	P-trend^1^	Q1	Q4	P-trend^1^	Q1	Q4	P-trend^1^
Person's general description of his/her sleep									
Cases	18	12		20	9		18	12	
Crude	1 (Ref.)	0.63 (0.29, 1.36)	0.13	1 (Ref.)	0.40 (0.18, 0.92)	0.02	1 (Ref.)	0.63 (0.29, 1.36)	0.11
Fully-adjusted model	1 (Ref.)	0.40 (0.09, 1.78)	0.13	1 (Ref.)	0.62 (0.18, 2.19)	0.22	1 (Ref.)	2.57 (0.53, 12.54)	0.36
Delay in falling asleep									
Cases	18	24		24	14		17	15	
Crude	1 (Ref.)	1.39 (0.72, 2.71)	0.29	1 (Ref.)	0.53 (0.26, 1.07)	0.06	1 (Ref.)	0.86 (0.41, 1.80)	0.52
Fully-adjusted model	1 (Ref.)	1.54 (0.49, 4.85)	0.47	1 (Ref.)	0.65 (0.22, 1.94)	0.27	1 (Ref.)	0.51 (0.15, 1.70)	0.26
Duration of beneficial sleep									
Cases	53	54		56	53		51	56	
Crude	1 (Ref.)	1.02 (0.62, 1.66)	0.70	1 (Ref.)	0.89 (0.55, 1.45)	0.78	1 (Ref.)	1.16 (0.71, 1.89)	0.31
Fully-adjusted model	1 (Ref.)	1.17 (0.56, 2.42)	0.40	1 (Ref.)	0.55 (0.27, 1.11)	0.07	1 (Ref.)	1.57 (0.73, 3.35)	0.35
Sleep efficiency^2^									
Cases	1	1		0	1		0	0	
Crude	1 (Ref.)	0.99 (0.06, 16.03)	0.99	1 (Ref.)	-	0.54	1 (Ref.)	-	0.99
Fully-adjusted model	1 (Ref.)	-	0.99	1 (Ref.)	-	0.99	1 (Ref.)	-	-
Sleep disorders									
Cases	13	16		16	13		12	15	
Crude	1 (Ref.)	1.25 (0.58, 2.72)	0.63	1 (Ref.)	0.78 (0.36, 1.69)	0.59	1 (Ref.)	1.27 (0.57, 2.83)	0.46
Fully-adjusted model	1 (Ref.)	1.26 (0.31, 5.15)	0.74	1 (Ref.)	0.73 (0.21, 2.53)	0.56	1 (Ref.)	1.03 (0.24, 4.48)	0.68
Use of sleeping medicine									
Cases	6	5		6	6		2	7	
Crude	1 (Ref.)	0.82 (0.24, 2.76)	0.66	1 (Ref.)	0.98 (0.31, 3.13)	0.84	1 (Ref.)	3.61 (0.74, 17.72)	0.21
Fully-adjusted model	1 (Ref.)	0.22 (0.03, 1.83)	0.14	1 (Ref.)	0.16 (0.01, 1.98)	0.06	1 (Ref.)	12.35 (0.86, 178.38)	0.05
Daily functional disorders									
Cases	20	14		19	13		16	16	
Crude	1 (Ref.)	0.66 (0.32, 1.37)	0.38	1 (Ref.)	0.64 (0.30, 1.35)	0.15	1 (Ref.)	0.99 (0.47, 2.08)	0.94
Fully-adjusted model	1 (Ref.)	0.50 (0.14, 1.86)	0.39	1 (Ref.)	1.46 (0.45, 4.72)	0.69	1 (Ref.)	4.55 (1.09, 18.91)	0.02

All values are odds ratios and 95% confidence intervals. Fully adjusted model: Adjusted for age, sex, energy intake, marital status, physical activity, homeownership, anti-depressing medication, education status, obstructive sleep apnea (OSA), distress, family size, history of hypertension, diabetes, smoking status, coffee and tea consumption, anxiety and BMI.

^1^P trend was obtained by considering the quartiles of majors NPs as ordinal variable.

^2^OR (95% CI) could not be calculated, due to not having cases in one quartile.

Crude and multivariate adjusted odds ratios (ORs) and 95% CIs for short sleeping and poor sleep quality across quartiles of different NPs, stratified by BMI categories, are presented in Table 6. Normal-weight participants in the forth quartile of NP2 (high vegetable), compared to the first one, were less likely to be short sleepers (OR 0.05, 95% CI 0.01, 0.37), after controlling all cofounders. However, higher adherence to NP3 was associated with higher odds of being short sleeper in normal-weight participants (OR 6.03, 95% CI 1.23, 29.57). With regard to sleep quality, higher adherence to NP2 was associated with lower odds of having poor sleep quality in normal-weight participants (OR 0.09, 95% CI 0.01, 0.62). No significant relationship was observed between sleep quality and NPs among overweight/obese adults in this analysis.

**Table 6:** Multivariate adjusted odds ratio (OR) and 95% confidence interval (CI) for short sleeping and poor quality of life across quartiles of major nutrient patterns (NPs), stratified by BMI categories.

	Quartiles of NP1 High animal proteins			Quartiles of NP2 High vegetable			Quartiles of NP3 High carbohydrate		
	Q1	Q4	P-trend^1^	Q1	Q4	P-trend^1^	Q1	Q4	P-trend^1^
Short sleeping									
Normal-weight (BMI < 25)									
Participants/cases	45/11	40/11		45/14	40/6		48/6	40/13	
Crude	1 (Ref.)	1.17 (0.44, 3.10)	0.29	1 (Ref.)	0.39 (0.13, 1.14)	0.23	1 (Ref.)	3.37 (1.14, 9.94)	0.01
Model 1	1 (Ref.)	1.43 (0.43, 4.79)	0.26	1 (Ref.)	0.22 (0.06, 0.76)	0.04	1 (Ref.)	3.57 (0.99, 12.93)	0.03
Model 2	1 (Ref.)	2.76 (0.53, 14.44)	0.08	1 (Ref.)	0.05 (0.01, 0.37)	0.01	1 (Ref.)	6.03 (1.23, 29.57)	0.03
Overweight and obese (BMI ≥ 25)									
Participants/cases	87/23	93/29		88/22	95/25		84/21	93/29	
Crude	1 (Ref.)	1.26 (0.66, 2.41)	0.55	1 (Ref.)	1.07 (0.55, 2.08)	0.92	1 (Ref.)	1.36 (0.70, 2.63)	0.26
Model 1	1 (Ref.)	0.97 (0.45, 2.08)	0.85	1 (Ref.)	0.68 (0.32, 1.46)	0.86	1 (Ref.)	1.10 (0.46, 2.60)	0.73
Model 2	1 (Ref.)	0.88 (0.31, 2.49)	0.96	1 (Ref.)	0.39 (0.14, 1.08)	0.03	1 (Ref.)	2.00 (0.64, 6.36)	0.33
Poor quality of sleep									
Normal-weight (BMI < 25)									
Participants/cases	45/14	40/11		45/12	40/8		48/12	40/14	
Crude	1 (Ref.)	0.84 (0.33, 2.15)	0.96	1 (Ref.)	0.69 (0.25, 1.90)	0.70	1 (Ref.)	1.62 (0.64, 4.06)	0.26
Model 1	1 (Ref.)	0.84 (0.27, 2.62)	0.96	1 (Ref.)	0.64 (0.20, 1.10)	0.71	1 (Ref.)	2.94 (0.91, 9.56)	0.06
Model 2	1 (Ref.)	2.50 (0.44, 14.24)	0.13	1 (Ref.)	0.09 (0.01, 0.62)	0.03	1 (Ref.)	3.20 (0.61, 16.77)	0.20
Overweight and obese (BMI ≥ 25)									
Participants/cases	87/27	93/31		88/33	95/23		84/18	93/27	
Crude	1 (Ref.)	1.11 (0.59, 2.08)	0.76	1 (Ref.)	0.53 (0.28, 1.01)	0.06	1 (Ref.)	1.50 (0.76, 2.98)	0.54
Model 1	1 (Ref.)	1.01 (0.48, 2.13)	0.95	1 (Ref.)	0.36 (0.17, 0.77)	0.01	1 (Ref.)	1.65 (0.68, 4.00)	0.49
Model 2	1 (Ref.)	0.74 (0.24, 2.27)	0.60	1 (Ref.)	0.56 (0.20, 1.61)	0.21	1 (Ref.)	1.05 (0.29, 3.88)	0.55

All values are odds ratios and 95% confidence intervals. Model 1: Adjusted for age, sex, and energy intake. Model 2: Additionally adjusted for marital status, physical activity, homeownership, anti-depressing medication, education status, obstructive sleep apnea (OSA), distress, family size, history of hypertension, diabetes, smoking status, coffee and tea consumption, and anxiety.

^1^Obtained by the use of quartiles of major nutrient patterns as an ordinal variable in the model.

Out of a total of 535 participants, 261 individuals (48.8%) had sufficient serum vitamin D levels (≥ 30 ng/mL), while 274 other participants (51.2%) had insufficient or deficient levels of serum vitamin D (< 30 ng/mL). Prevalence of vitamin D insufficiency or deficiency was higher in those with the most adherence to NP3 compared to those with the less adherence (Q4 vs. Q1: 59.4 vs. 40.9%; P = 0.03). Prevalence of vitamin D insufficiency or deficiency was not significantly different in categories of NP1 or NP2. Additionally, prevalence of vitamin D sufficiency was not significantly different among sleep quality or quantity levels (data not shown).

Crude and multivariate adjusted odds ratios (ORs) and 95% CIs for short sleeping and poor sleep quality across categories of NP2 and 3 interacted with vitamin D deficiency are illustrated in Table 7. Based on above-mentioned results of the study, a significant decreased odds were observed between adherence of NP2 and poor sleep quality and short sleeping. Therefore, NP2 (high vegetable) was considered as a healthy NP. Also, a direct relation among adherence of NP3 and poor sleep quality and short sleeping was found. Therefore, NP3 (high carbohydrate) was considered as an unhealthy NP. The relation between NP1 and sleep quality and short sleeping was not clear; so, this pattern was not included in the interaction analysis of NPs with vitamin D status. Participants with low adherence to NP2 and insufficient/deficient level of vitamin D, compared to those with more adherence to NP2 and sufficient vitamin D level, had a higher chance of being short sleepers (OR 3.42, 95% CI 1.42, 6.64). However, there was no significant interaction between this NP (NP2) and circulating levels of vitamin D with sleep quality. Also, no significant association was observed between adherence of NP3 and sleep quality or quantity, considering interactions with vitamin D status.

**Table 7:** Multivariate adjusted odds ratio (OR) and 95% confidence interval (CI) for short sleeping and poor quality of life across categories NP 2 and 3 interacted with vitamin D deficiency.

NP2					
	Healthy NP and sufficient vitamin D	Healthy NP and insufficient or deficient vitamin D	Unhealthy NP and sufficient vitamin D	Unhealthy NP and insufficient or deficient vitamin D	P-trend^1^
Short sleeping					
Participants/cases (n)	65/16	70/15	65/15	68/21	
Crude	1 (Ref.)	0.91 (0.52, 1.58)	0.97 (0.56, 1.69)	1.09 (0.64, 1.89)	0.68
Model 1	1 (Ref.)	0.85 (0.48, 1.51)	1.28 (0.71, 2.32)	1.51 (0.83, 2.73)	0.12
Model 2	1 (Ref.)	0.99 (0.47, 2.09)	2.12 (0.97, 4.63)	3.12 (1.45, 6.73)	0.002
Model 3	1 (Ref.)	0.99 (0.47, 2.11)	2.13 (0.97, 4.66)	3.07 (1.42, 6.64)	0.002
Poor quality of sleep					
Participants/Cases (n)	65/18	70/13	65/18	68/27	
Crude	1 (Ref.)	0.69 (0.40, 1.19)	0.87 (0.51, 1.48)	1.19 (0.71, 2.00)	0.35
Model 1	1 (Ref.)	0.74 (0.42, 1.28)	1.02 (0.58, 1.80)	1.44 (0.82, 2.52)	0.15
Model 2	1 (Ref.)	0.48 (0.23, 1.02)	0.82 (0.37, 1.82)	1.13 (0.54, 2.35)	0.54
Model 3	1 (Ref.)	0.47 (0.22, 1.01)	0.83 (0.37, 1.83)	1.15 (0.55, 2.39)	0.51

NP3					
	Healthy NP and sufficient vitamin D	Healthy NP and insufficient or deficient vitamin D	Unhealthy NP and sufficient vitamin D	Unhealthy NP and insufficient or deficient vitamin D	P-trend^1^
Short sleeping					
Participants/cases (n)	54/20	79/22	78/17	54/10	
Crude	1 (Ref.)	1.04 (0.58, 1.89)	1.79 (1.02, 3.15)	1.63 (0.95, 2.79)	0.03
Model 1	1 (Ref.)	1.09 (0.60, 1.99)	1.70 (0.92, 3.16)	1.54 (0.84, 2.83)	0.13
Model 2	1 (Ref.)	1.12 (0.53, 2.34)	1.49 (0.67, 3.30)	1.96 (0.93, 4.15)	0.06
Model 3	1 (Ref.)	1.10 (0.53, 2.31)	1.46 (0.66, 3.24)	1.92 (0.91, 4.06)	0.07
Poor quality of sleep					
Participants/cases (n)	54/24	79/17	78/18	54/12	
Crude	1 (Ref.)	1.26 (0.74, 2.17)	1.47 (0.86, 2.51)	1.12 (0.66, 1.88)	0.06
Model 1	1 (Ref.)	1.35 (0.78, 2.34)	1.62 (0.89, 2.95)	1.28 (0.71, 2.31)	0.39
Model 2	1 (Ref.)	1.14 (0.55, 2.34)	1.40 (0.62, 3.18)	0.91 (0.41, 2.02)	0.84
Model 3	1 (Ref.)	1.14 (0.56, 2.35)	1.43 (0.63, 3.25)	0.93 (0.42, 2.06)	0.87

All values are odds ratios and 95% confidence intervals. Healthy NP refers to high adherence to NP2 (quartile 3 and 4 of this NP) and low adherence to NP2 (quartile 1 and 2 of this NP). Sufficient serum vitamin D refers to 25(OH)D ≥ 30 ng/ml and insufficient or deficient levels of serum vitamin D refers to 25(OH)D < 30 ng/ml. Model 1: Adjusted for age, sex, and energy intake. Model 2: Additionally adjusted for marital status, physical activity, homeownership, anti-depressing medication, education status, obstructive sleep apnea (OSA), distress, family size, history of hypertension, diabetes, smoking, coffee and tea consumption, and anxiety; Model 3: Additionally, adjusted for BMI.

## Discussion

The current cross-sectional study revealed that higher adherence to a high vegetable NP was inversely associated with short sleeping and poor sleep quality, whereas higher adherence to NP of high carbohydrate increased the likelihood of short sleeping, particularly in Iranian adults with normal weight. In addition, individuals with low adherence to NP2 (vegetable consumption) and insufficient/deficient vitamin D levels had a greater likelihood of being short sleepers. To our knowledge, this is the first epidemiologic study that evaluated the linkage between nutrient patterns and sleep duration and sleep quality in Iranian adults, in addition tried to assess this relation across categories of NPs interacted with vitamin D status.

Sleep disorders could elevate vulnerability to adverse health outcomes. Sleep disorders might increase morbidity to cardio-metabolic diseases such as hypertension^55^, diabetes^56^, depression^57^, and mortality^14–16^. Therefore, it would be suggested that dietitians would advise adults to increase their fiber, potassium, vitamin A, C, K, and other nutrients of vegetables, decrease their carbohydrate-related nutrients, and improve their serum vitamin D levels in order to reduce the risk of sleep disturbances. In other words, the Iranian population might be encouraged to consume foods with a higher proportion of complex carbohydrates and fiber, such as green leafy vegetables, and whole grain and a lower proportion of simple carbohydrates, such as starchy vegetables and refined grains.

NP1 in the current study was labeled as "high animal protein". This pattern was rich in animal protein, cobalamin, zinc, SFA, phosphorus, riboflavin, cholesterol, MUFAs, calcium, and pantothenic acid. Participants in the highest category of high animal protein pattern consumed higher red and processed meat, SFA, dairy, white meat, and energy than the first category. High intake of animal protein and cobalamin, which was especially originated from animal meat, in combination with moderate intake of SFA, cholesterol, and phosphorous could result in a Western dietary pattern among Iranian adults. A systematic review of dietary patterns documented that meat consumption could increase the odds of sleep disorders (such as poor sleep quality, short sleeping, and insomnia)^58^. The interactions between sleep disorder-inducing nutrients such as red and processed meat and SFA^59^ and sleep disorder-protective nutrients such as zinc^60^ and cobalamin^59^ might result in a non-significant relation between NP1 and sleep duration or sleep quality in the current investigation.

NP2 in the present investigation was characterized by high consumption of fiber, vitamin C, K, B9, A, B6, TFA, magnesium, and copper. We labeled this NP as "high vegetable" pattern. Since nutrients in vegetables such as dietary fiber, vitamin C and K had high loadings in this NP. Our findings were consistent with previous observational investigations that suggested inverse associations between high vegetable and sufficient levels of vitamin D with decreased odds of short sleeping and lower sleep quality. For instance, Pengpid et al. found a 49% decreased in odds of poor sleep quality and a 33% lower chance of short sleeping among university students of 28 countries who consume 3 servings/day fruits and vegetables compared to those who ate 0–1 serving/day^61^. A recent clinical trial on young adults with a mean age of 26.0 (± 2.8) years also revealed that women who increased their fruits and vegetables intake > 3 servings/day for 3 months had a 0.2-point higher sleep quality score in comparison to control group^62^. A meta-analysis of three randomized clinical trials (RCTs) revealed that vitamin D supplementation had no significant effect on sleep quality (defined by PSQI)^63^. However, the meta-analysis of four pre-post studies with vitamin D supplementation for 8 to 12 weeks revealed a significant beneficial impact of intervention on sleep quality (mean difference: − 2.33; 95% CI − 3.09, − 1.57)^63^. Another meta-analysis on observational investigation included 9397 adults showed that vitamin D deficiency (~ 23 ng/mL) could increase the chance of both short sleeping (OR 1.74, 95% CI 1.30, 2.32) and poor sleep quality (OR 1.59, 95% CI 1.23, 2.05)^64^. In the current cross-sectional study, low adherence to high vegetable pattern and vitamin D deficiency, compared with high adherence to this pattern and sufficient level of vitamin D, were synergistically associated with increased odds of short sleeping. There might also be a U-shaped relationship between circulating levels of vitamin D and short sleeping. An RCT with a cross-over design on young adults, compared three different dietary patterns (high fat, carbohydrate, and protein) on sleep quality^65^, showed that higher adherence to the high carbohydrate pattern had a significant connection with short sleeping (defined by PSQI). This intervention also indicated that administration of the high fat dietary pattern was related to a significant lower odds of poor sleep quality in comparison to other dietary patterns^65^.

Vegetables contain a high content of melatonin and serotonin, which could explain our findings^66^. In addition, vegetable antioxidants may reduce oxidative stress and consequently enhance sleep quality^67^. Vitamin D bind receptors in brain-stem areas involve in sleep regulation; moreover, vitamin D can regulate tryptophan conversion into 5-hydroxytryptophan, which expresses vitamin D response element (VDRE) at the gene level. In turn, 5-hydroxytryptophan is metabolized to serotonin to produce the sleep hormone, melatonin^34^. Despite the fact that the melatonin content of vegetables is well-known, the bioavailability is a matter of controversy, since other dietary components may alter melatonin absorption by the gastrointestinal tract or its metabolism in various organs^68^.

Several strengths of this study should be highlighted. This is the first study among Iranian adults which examined dietary nutrient patterns and circulating 25-hydroxy vitamin D with sleep outcomes. A multistage cluster random sampling technique was utilized to have a relatively representative sample of Iranian adults. We acknowledge several limitations of present study, as well. First, a casual relation between dietary nutrient patterns and vitamin D levels with sleep outcomes cannot be inferred from this cross-sectional study. Some degree of error in dietary assessment was inevitable due to recall bias or other possible reporting biases. Moreover, although our data collection was not conducted during COVID-19 waves or quarantine time, this pandemic might change the food intake, sleep habits and circulating vitamin D level of the population^69^ and therefore, might affect our findings. Finally, although several potential variables were taken into account in the analyses, residual confounding cannot be ruled out.

In conclusion, this population-based, cross-sectional study revealed that high adherence to pattern comprising mainly of vegetable nutrients was associated with a reduced likelihood of being short sleepers and having poor sleep quality in Iranian adults, especially among those with a normal weight. Lower adherence to vegetable NP and insufficient/deficient vitamin D levels were synergistically associated with greater likelihood of being short sleepers. Greater adherence to carbohydrate NP was associated with an increased likelihood of short sleeping.

### Supplementary Information

Supplementary Figure S1.

## Data Availability

The data that support the findings of this study are available from the corresponding author [PS], upon reasonable request.

## Abbreviations

ANOVA: Analysis of variance
ANCOVA: Analysis of covariance
NSF: National Sleep Foundation
IBD: Inflammatory bowel diseases
VDD: Vitamin D deficiency
WC: Waist circumference
95% CI: 95% Confidence interval
OSA: Obstructive sleep apnea
WHO: World Health Organization
OR: Odd ratio
FBG: Fasting blood glucose
SD: Standard deviation
SE: Standard error
FFQ: Food frequency questionnaire
SPSS: Statistical package for the social sciences
VDRE: Vitamin D response element
VDS: Vitamin D supplementation
RCT: Randomized clinical trial
BMI: Body mass index

## References

[1] Dahl RE (1996). The regulation of sleep and arousal: Development and psychopathology. Dev. Psychopathol..

[2] Hirshkowitz M, Whiton K, Albert SM, Alessi C, Bruni O, DonCarlos L (2015). National Sleep Foundation’s sleep time duration recommendations: Methodology and results summary. Sleep Health.

[3] Kerkhof GA (2017). Epidemiology of sleep and sleep disorders in The Netherlands. Sleep Med..

[4] Harding K, Feldman M (2008). Sleep disorders and sleep deprivation: An unmet public health problem. J. Am. Acad. Child Adolesc. Psychiatry.

[5] Léger D, Poursain B, Neubauer D, Uchiyama M (2008). An international survey of sleeping problems in the general population. Curr. Med. Res. Opin..

[6] Stranges S, Tigbe W, Gómez-Olivé FX, Thorogood M, Kandala N-B (2012). Sleep problems: An emerging global epidemic? Findings from the INDEPTH WHO-SAGE study among more than 40,000 older adults from 8 countries across Africa and Asia. Sleep.

[7] Alexander M, Ray MA, Hébert JR, Youngstedt SD, Zhang H, Steck SE (2016). The national veteran sleep disorder study: Descriptive epidemiology and secular trends, 2000–2010. Sleep.

[8] Hoyos C, Glozier N, Marshall NS (2015). Recent evidence on worldwide trends on sleep duration. Curr. Sleep Med. Rep..

[9] Jin Q, Yang N, Dai J, Zhao Y, Zhang X, Yin J, Yan Y (2022). Association of sleep duration with all-cause and cardiovascular mortality: A prospective cohort study. Front. Public Health.

[10] Krueger PM, Friedman EM (2009). Sleep duration in the United States: A cross-sectional population-based study. Am. J. Epidemiol..

[11] Park S, Cho MJ, Chang SM, Bae JN, Jeon HJ, Cho SJ (2010). Relationships of sleep duration with sociodemographic and health-related factors, psychiatric disorders and sleep disturbances in a community sample of Korean adults. J. Sleep Res..

[12] Tucker P, Bejerot E, Kecklund G, Aronsson G, Åkerstedt T (2015). The impact of work time control on physicians' sleep and well-being. Appl. Ergon..

[13] Lewis PA, Knoblich G, Poe G (2018). How memory replay in sleep boosts creative problem-solving. Trends Cogn. Sci..

[14] Åkerstedt T, Ghilotti F, Grotta A, Zhao H, Adami HO, Trolle-Lagerros Y (2019). Sleep duration and mortality: Does weekend sleep matter?. J. Sleep Res..

[15] Guglielmi O, Lanteri P, Garbarino S (2019). Association between socioeconomic status, belonging to an ethnic minority and obstructive sleep apnea: A systematic review of the literature. Sleep Med..

[16] Ash T, Davison KK, Haneuse S, Horan C, Kitos N, Redline S (2019). Emergence of racial/ethnic differences in infant sleep duration in the first six months of life. Sleep Med. X..

[17] Hoevenaar-Blom MP, Spijkerman AMW, Kromhout D, van den Berg JF, Verschuren WMM (2011). Sleep duration and sleep quality in relation to 12-year cardiovascular disease incidence: The MORGEN study. Sleep.

[18] Okun ML, Mancuso RA, Hobel CJ, Schetter CD, Coussons-Read M (2018). Poor sleep quality increases symptoms of depression and anxiety in postpartum women. J. Behav. Med..

[19] Gildner TE, Liebert MA, Kowal P, Chatterji S, Josh SJ (2014). Sleep duration, sleep quality, and obesity risk among older adults from six middle-income countries: Findings from the study on global AGEing and adult health (SAGE). Am. J. Hum. Biol..

[20] Binks H, Vincent G, Gupta C, Irwin C, Khalesi S (2020). Effects of diet on sleep: A narrative review. Nutrients.

[21] Jahrami H, BaHammam AS, Bragazzi NL, Saif Z, Faris M, Vitiello MV (2021). Sleep problems during the COVID-19 pandemic by population: A systematic review and meta-analysis. J. Clin. Sleep Med..

[22] Xie Y, Liu S, Chen XJ, Yu HH, Yang Y, Wang W (2021). Effects of exercise on sleep quality and insomnia in adults: A systematic review and meta-analysis of randomized controlled trials. Front. Psychiatry.

[23] Melzer K, Kayser B, Saris WH, Pichard C (2005). Effects of physical activity on food intake. Clin. Nutr..

[24] Katagiri R, Asakura K, Kobayashi S, Suga H, Sasaki S (2014). Low intake of vegetables, high intake of confectionary, and unhealthy eating habits are associated with poor sleep quality among middle-aged female Japanese workers. J. Occup. Health.

[25] Grandner MA, Kripke DF, Naidoo N, Langer RD (2010). Relationships among dietary nutrients and subjective sleep, objective sleep, and napping in women. Sleep Med..

[26] Grandner MA, Jackson N, Gerstner JR, Knutson KL (2014). Sleep symptoms associated with intake of specific dietary nutrients. J. Sleep Res..

[27] Norman AW (2008). From vitamin D to hormone D: Fundamentals of the vitamin D endocrine system essential for good health. Am. J. Clin. Nutr..

[28] Wierzbicka A, Oczkowicz M (2022). Sex differences in vitamin D metabolism, serum levels and action. Br. J. Nutr..

[29] Mozos I, Marginean O (2015). Links between vitamin D deficiency and cardiovascular diseases. BioMed Res. Int..

[30] Holick MF, Chen TC (2008). Vitamin D deficiency: A worldwide problem with health consequences. Am. J. Clin. Nutr..

[31] McCarty DE, Chesson AL, Jain SK, Marino AA (2014). The link between vitamin D metabolism and sleep medicine. Sleep Med. Rev..

[32] Evatt ML (2015). Vitamin D associations and sleep physiology—promising rays of information. Sleep.

[33] Archontogeorgis K, Nena E, Steiropoulos P (2020). Linking Vitamin D and Sleep.

[34] Muscogiuri G, Barrea L, Scannapieco M, Di Somma C, Scacchi M, Aimaretti G (2019). The lullaby of the sun: The role of vitamin D in sleep disturbance. Sleep Med..

[35] Muzur A, Pace-Schott EF, Hobson JA (2002). The prefrontal cortex in sleep. Trends Cogn. Sci..

[36] Willett W (2012). Nutritional Epidemiology.

[37] Malmir H, Shayanfar M, Mohammad-Shirazi M, Tabibi H, Sharifi G, Esmaillzadeh A (2019). Patterns of nutrients intakes in relation to glioma: A case-control study. Clin. Nutr..

[38] Freisling H, Fahey MT, Moskal A, Ocké MC, Ferrari P, Jenab M (2010). Region-specific nutrient intake patterns exhibit a geographical gradient within and between European countries. J Nutr..

[39] Beebe D, Chang JJ, Kress K, Mattfeldt-Beman M (2017). Diet quality and sleep quality among day and night shift nurses. J. Nurs. Manag..

[40] Theorell-Haglöw J, Lemming EW, Michaëlsson K, Elmståhl S, Lind L, Lindberg E (2020). Sleep duration is associated with healthy diet scores and meal patterns: Results from the population-based EpiHealth study. J. Clin. Sleep Med..

[41] Castro-Diehl C, Wood AC, Redline S, Reid M, Johnson DA, Maras JE (2018). Mediterranean diet pattern and sleep duration and insomnia symptoms in the Multi-Ethnic Study of Atherosclerosis. Sleep..

[42] Godos J, Ferri R, Caraci F, Cosentino FII, Castellano S, Galvano F (2019). Adherence to the mediterranean diet is associated with better sleep quality in Italian adults. Nutrients.

[43] van der Merwe C, Münch M, Kruger R (2022). Chronotype differences in body composition, dietary intake and eating behavior outcomes: A scoping systematic review. Adv. Nutr..

[44] Norouzi M, Hosseini B, Yaseri M, Heydari Araghi M, Omidian K, Djfarian K (2018). The association between sleep pattern and nutrients intake pattern in healthy overweight and obese adults. Sleep.

[45] Dauvilliers Y, Evangelista E, Lopez R, Barateau L, Scholz S, de Paulet BC (2017). Vitamin D deficiency in type 1 narcolepsy: A reappraisal. Sleep Med..

[46] Piovezan RD, Hirotsu C, Feres MC, Cintra FD, Andersen ML, Tufik S (2017). Obstructive sleep apnea and objective short sleep duration are independently associated with the risk of serum vitamin D deficiency. PLoS ONE.

[47] Shiue I (2013). Low vitamin D levels in adults with longer time to fall asleep: US NHANES, 2005–2006. Int. J. Cardiol..

[48] Haghighatdoost F, Karimi G, Esmaillzadeh A, Azadbakht L (2012). Sleep deprivation is associated with lower diet quality indices and higher rate of general and central obesity among young female students in Iran. Nutrients.

[49] Mirmiran P, Esfahani FH, Mehrabi Y, Hedayati M, Azizi F (2010). Reliability and relative validity of an FFQ for nutrients in the Tehran lipid and glucose study. Public Health Nutr..

[50] Ghaffarpour M, Houshiar-Rad A, Kianfar H (1999). The manual for household measures, cooking yields factors and edible portion of foods. Tehran Nashre Olume Keshavarzy..

[51] Buysse DJ, Reynolds CF, Monk TH, Berman SR, Kupfer DJ (1989). The Pittsburgh Sleep Quality Index: A new instrument for psychiatric practice and research. Psychiatry Res..

[52] Heidary A, Marashi M (2010). Association between insomnia, sleep quality, drowsiness, mental health with academic performance in gairls. J. Women Cult..

[53] Castro-Diehl C, Wood AC, Redline S, Reid M, Johnson DA, Maras JE, Jacobs DR, Shea S, Crawford A, St-Onge MP (2018). Mediterranean diet pattern and sleep duration and insomnia symptoms in the Multi-Ethnic Study of Atherosclerosis. Sleep.

[54] Campanini MZ, Guallar-Castillón P, Rodríguez-Artalejo F, Lopez-Garcia E (2017). Mediterranean diet and changes in sleep duration and indicators of sleep quality in older adults. Sleep.

[55] Lo K, Woo B, Wong M, Tam W (2018). Subjective sleep quality, blood pressure, and hypertension: A meta-analysis. J. Clin. Hypertens..

[56] Cappuccio FP, Cooper D, D'Elia L, Strazzullo P, Miller MA (2011). Sleep duration predicts cardiovascular outcomes: A systematic review and meta-analysis of prospective studies. Eur. Heart J..

[57] Patel SR, Zhu X, Storfer-Isser A, Mehra R, Jenny NS, Tracy R (2009). Sleep duration and biomarkers of inflammation. Sleep.

[58] Godos J, Grosso G, Castellano S, Galvano F, Caraci F, Ferri R (2021). Association between diet and sleep quality: A systematic review. Sleep Med. Rev..

[59] Zhao M, Tuo H, Wang S, Zhao L (2020). The effects of dietary nutrition on sleep and sleep disorders. Mediat. Inflamm..

[60] Saito H, Cherasse Y, Suzuki R, Mitarai M, Ueda F, Urade Y (2017). Zinc-rich oysters as well as zinc-yeast-and astaxanthin-enriched food improved sleep efficiency and sleep onset in a randomized controlled trial of healthy individuals. Mol. Nutr. Food Res..

[61] Pengpid S, Peltzer K (2020). Fruit and vegetable consumption is protective from short sleep and poor sleep quality among university students from 28 countries. Nat. Sci. Sleep.

[62] Jansen EC, She R, Rukstalis M, Alexander GL (2021). Changes in fruit and vegetable consumption in relation to changes in sleep characteristics over a 3-month period among young adults. Sleep Health.

[63] Abboud M (2022). Vitamin D supplementation and sleep: A systematic review and meta-analysis of intervention studies. Nutrients.

[64] Gao Q, Kou T, Zhuang B, Ren Y, Dong X, Wang Q (2018). The association between vitamin d deficiency and sleep disorders: A systematic review and meta-analysis. Nutrients.

[65] Lindseth G, Murray A (2016). Dietary macronutrients and sleep. West. J. Nurs. Res..

[66] Meng X, Li Y, Li S, Zhou Y, Gan R-Y, Xu D-P (2017). Dietary sources and bioactivities of melatonin. Nutrients.

[67] St-Onge MP, Mikic A, Pietrolungo CE (2016). Effects of diet on sleep quality. Adv. Nutr..

[68] Reiter RJ, Tan D-X, Manchester LC, Simopoulos AP, Maldonado MD, Flores LJ (2007). Melatonin in edible plants (phytomelatonin): Identification, concentrations, bioavailability and proposed functions. World Rev. Nutr. Diet..

[69] Alharbi HF, Barakat H (2022). Effect of COVID-19 pandemic on dietary habits and sleep quality applying the Pittsburgh sleep quality index in adult Saudi population: A cross-sectional study. Int. J. Environ. Res. Public Health.

