# Supplementary material for: Association between patterns of nutrient intake and circulating vitamin D with sleep status among Iranian adults
Source: Sci Rep. 2023 Sep 15;13:15318. doi: 10.1038/s41598-023-42661-6 (PMC10504293; doi:10.1038/s41598-023-42661-6)
Supplement: Supplementary file 1 — Supplementary Figure S1. [file 41598_2023_42661_MOESM1_ESM.pptx]

## Slide 1
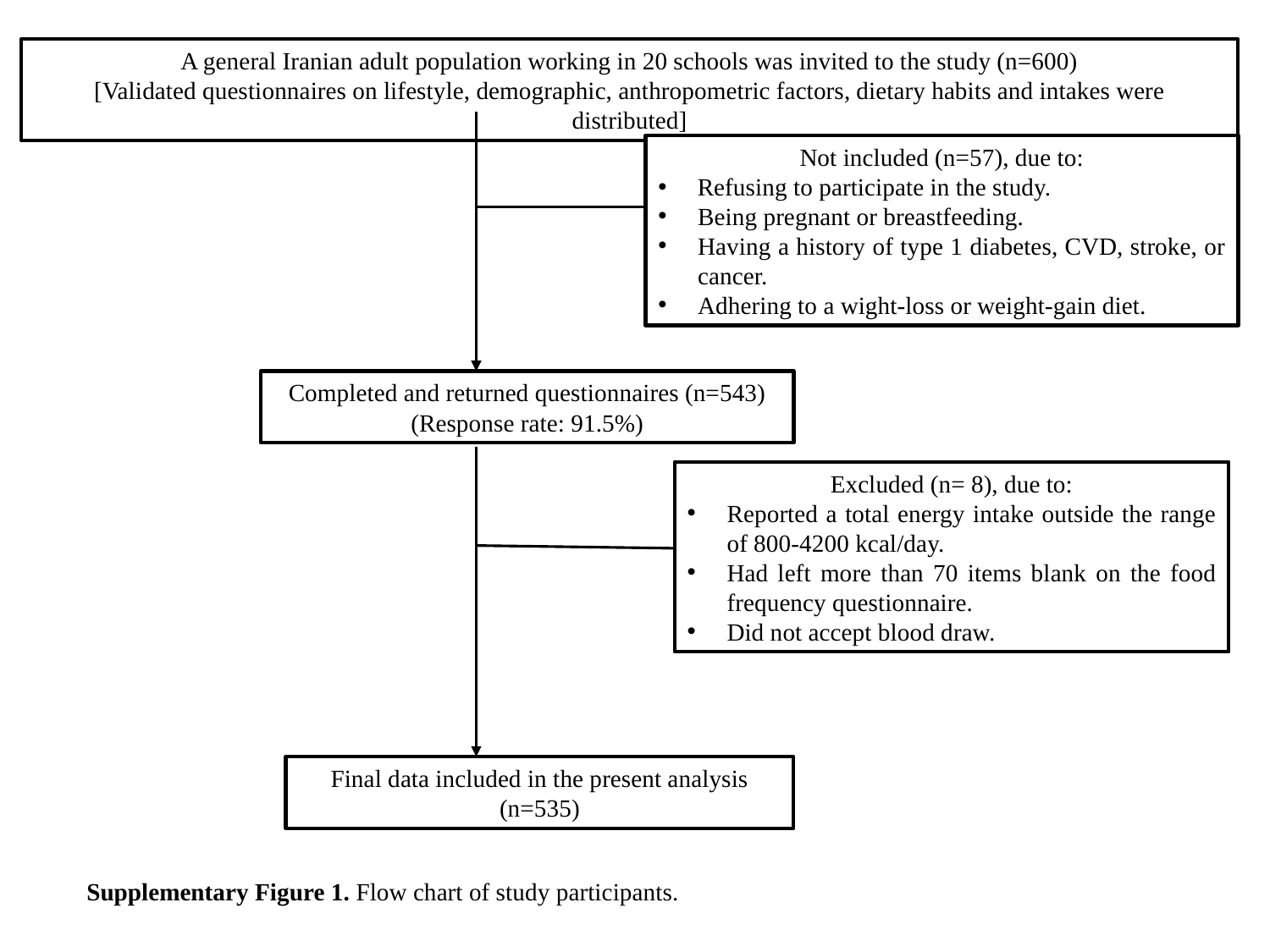

A general Iranian adult population working in 20 schools was invited to the study (n=600)
[Validated questionnaires on lifestyle, demographic, anthropometric factors, dietary habits and intakes were distributed]
Not included (n=57), due to:
 Refusing to participate in the study.
Being pregnant or breastfeeding.
Having a history of type 1 diabetes, CVD, stroke, or cancer.
Adhering to a wight-loss or weight-gain diet.
Completed and returned questionnaires (n=543)
(Response rate: 91.5%)
Excluded (n= 8), due to:
Reported a total energy intake outside the range of 800-4200 kcal/day.
Had left more than 70 items blank on the food frequency questionnaire.
Did not accept blood draw.
Final data included in the present analysis (n=535)
Supplementary Figure 1. Flow chart of study participants.
